# RETRACTION: Health lipid indices and physicochemical properties of dual fortified yogurt with extruded flaxseed omega fatty acids and fibers for hypercholesterolemic subjects

**DOI:** 10.1002/fsn3.4092

**Published:** 2024-04-05

## Abstract

**RETRACTION:** Nazir Ahmad, Muhammad Faisal Manzoor, Umair Shabbir, Saeed Ahmed, Tariq Ismail, Farhan Saeed, MahrUn Nisa, Faqir Muhammad Anjum, and Shahzad Hussain, “Health lipid indices and physicochemical properties of dual fortified yogurt with extruded flaxseed omega fatty acids and fibers for hypercholesterolemic subjects,” *Food Science & Nutrition* 8 (2020): 273–280, https://doi.org/10.1002/fsn3.1302.

The above article, published online on 26 December 2019 in Wiley Online Library (wileyonlinelibrary.com), has been retracted by agreement between the journal's Editor‐in‐Chief, Y. Martin Lo, and Wiley Periodicals, LLC. The retraction has been agreed to following concerns about the peer review process. The editorial office found unambiguous evidence that the manuscript was accepted solely based on compromised and insufficient reviewer reports. This evidence was further investigated and verified by the publisher and the Editor‐in‐Chief. As a result, the conclusions of this article are not considered reliable. The authors were notified of the retraction and disagreed with this decision.

**RETRACTION:** Nazir Ahmad, Muhammad Faisal Manzoor, Umair Shabbir, Saeed Ahmed, Tariq Ismail, Farhan Saeed, MahrUn Nisa, Faqir Muhammad Anjum, and Shahzad Hussain, “Health lipid indices and physicochemical properties of dual fortified yogurt with extruded flaxseed omega fatty acids and fibers for hypercholesterolemic subjects,” *Food Science & Nutrition* 8 (2020): 273–280, https://doi.org/10.1002/fsn3.1302.

The above article, published online on 26 December 2019 in Wiley Online Library (wileyonlinelibrary.com), has been retracted by agreement between the journal's Editor‐in‐Chief, Y. Martin Lo, and Wiley Periodicals, LLC. The retraction has been agreed to following concerns about the peer review process. The editorial office found unambiguous evidence that the manuscript was accepted solely based on compromised and insufficient reviewer reports. This evidence was further investigated and verified by the publisher and the Editor‐in‐Chief. As a result, the conclusions of this article are not considered reliable. The authors were notified of the retraction and disagreed with this decision.

